# Distributed Passive Positioning and Sorting Method for Multi-Network Frequency-Hopping Time Division Multiple Access Signals

**DOI:** 10.3390/s24227168

**Published:** 2024-11-08

**Authors:** Jiaqi Mao, Feng Luo, Xiaoquan Hu

**Affiliations:** 1 National Key Laboratory of Radar Signal Processing, Xidian University, Xi’an 710071, China; mjq2020xidian@163.com; 2 Xi’an Research Institute of Navigation Technology, Xi’an 710068, China; hjt910614@163.com

**Keywords:** TDMA signal, passive positioning, network stations sorting, cross ambiguity function (CAF), improved K-means method

## Abstract

When there are time division multiple access (TDMA) signals with large bandwidth, waveform aliasing, and fast frequency-hopping in space, current methods have difficulty achieving the accurate localization of radiation sources and signal-sorting from multiple network stations. To solve the above problems, a distributed passive positioning and network stations sorting method for broadband frequency-hopping signals based on two-level parameter estimation and joint clustering is proposed in this paper. Firstly, a two-stage filtering structure is designed to achieve control filtering for each frequency point. After narrowing down the parameter estimation range through adaptive threshold detection, the time difference of arrival (TDOA) and the velocity difference of arrival (VDOA) can be obtained via coherent accumulating based on the cross ambiguity function (CAF). Then, a multi-station positioning method based on the TDOA/VDOA is used to estimate the position of the target. Finally, the distributed joint eigenvectors of the multi-stations are constructed, and the signals belonging to different network stations are effectively classified using the improved K-means method. Numerical simulations indicate that the proposed method has a better positioning and sorting effect in low signal-to-noise (SNR) and low snapshot conditions compared with current methods.

## 1. Introduction

A frequency-hopping time division multiple access (TDMA) signal is a wireless data broadcast network using the TDMA access method. Members take turns occupying time slots to broadcast messages and receive messages sent by other members when they are not broadcasting. Frequency-hopping TDMA uses anti-jamming measures such as pulse-to-pulse frequency hopping within the time slots; therefore, it has extremely high confidentiality [1,2]. Moreover, frequency-hopping TDMA can allocate different transmit members in different networks. The network capacity is extended by changing the frequency-hopping pattern. Currently, frequency-hopping TDMA signals are widely carried on main combat platforms such as unmanned aerial vehicle (UAV) formations, which are typical targets that positioning systems need to quickly assess [3].

Modern warfare has crucial requirements for secrecy and concealment. Distributed passive localization technology has been proven to be effective against group targets [4]. Through inter-station data fusion processing, there is a high probability that reception and accurate localization of radiated source signals can be achieved. However, the frequency-hopping TDMA network cluster has a dynamically changing network topology, and the pseudo-random and high-speed hopping frequencies result in the severe aliasing of the received signals. It is very difficult for existing localization systems to discover and locate targets accurately with high probability, and it is also difficult to identify the network structure and the relationship between grouped targets.

To simultaneously estimate the position and velocity of a target, researchers have proposed a localization method based on the TDOA and VDOA [5,6,7]. As we know, the positioning effect of frequency-hopping signals mainly depends on the accuracy of the parameter estimation [8]. In [9,10,11], the reception time was divided into short time periods, and the maximum likelihood estimation (MLE) of the time delay at the observation starting point and the Doppler frequency shift at the signal carrier frequency were calculated. By compensating for the offset, a higher estimation accuracy was obtained. However, due to the short duration of the short-term segments and the non-coherent nature of the fusion, the accuracy of the parameter estimation was low. In addition, the TDOA estimation method and the envelope fitting method for the correlation function of a single-hop signal were proposed in [12]. This method performs a precise small-scale search near the relevant main peak, which can reduce the estimation error of the TDOA at low SNR. However, this method is only applicable to a narrowband model. In [13,14], the TDOA and the VDOA estimation algorithm based on the CAF for multi-hop signal coherent accumulation was proposed. This method normalizes the frequency difference of each hop signal. However, the estimation of the TDOA will be affected by periodic peaks and has a significant threshold effect. In [15], the stepwise accuracy enhancement (SAE) method was proposed. This method describes the joint parameter estimation as a quadratic constrained quadratic program (QCQP) problem. The estimation bias of the parameters is reduced by minimizing the impact of the TDOA measurement error. This method can improve the accuracy of far-field positioning, but it is prone to converge to local optimal solutions and is greatly affected by noise.

To achieve the sorting of signals from different network stations, researchers have proposed two effective methods. One is a sorting method based on blind source separation, and the other is a sorting method based on spatiotemporal frequency information extraction. In [16], a method for sorting signals using polarization frequency correlation was proposed. However, the performance of this method is affected by SNR and channel variability. In [17], a feature-set classification method for a time–frequency graph of connected domains based on improved K-means clustering was proposed. However, this method cannot achieve the correlation of jump signals when there is a multipath effect in the arrival time and in the angle of the signal. In [18], the original objective function was reformulated as a trace maximization problem to optimize the K-means algorithm. In addition, a balanced fair K-means clustering algorithm was presented in [19]. These two methods improved the clustering accuracy to a certain extent but could not solve the problem of local convergence. In [20,21,22,23], a sorting method based on convolutional neural networks (CNNs) and recurrent neural networks (RNNs) was proposed, which can reduce the impact of the time–frequency resolution and the spectral leakage on the signal sorting accuracy. However, the computational complexity of the algorithm is high, and it requires a large amount of data to be trained offline with poor real-time performance. In [24], a time–frequency mixed estimation method based on density peak clustering and tensor decomposition was proposed. By concatenating different segments of signals based on angles, frequency-hopping signals can be sorted in the presence of time–frequency overlap. However, the accuracy of the angle measurement seriously affects the sorting effect of the frequency-hopping signals, and this method does not consider the situation of multiple network stations. In [25], a signal sorting method based on Bayesian architecture was proposed. The method uses a dynamic cluster merging (DCM) algorithm to merge clusters of the same type of signals. It has a high sorting accuracy when the PDW varies massively and the number of signals changes dynamically over time. However, this method is unable to distinguish between different targets within the same network cluster.

In this paper, we propose a distributed passive positioning and multi-network signal sorting method suitable for frequency-hopping TDMA signals. This method follows four steps. The first step is to achieve the control filtering of each frequency point of the TDMA signal through two-stage filtering. The second step is to narrow down the parameter search interval through an adaptive threshold and then achieve an accurate and unbiased estimation of the TDOA and VDOA parameters based on a small number of samples. The third step is to calculate the position of the radiation source based on the parameter estimation values and to construct a joint feature vector sample set. The fourth step is to divide the radiation source feature set into different communication networks based on the degree of correlation between samples. This method can effectively improve the accuracy of the signal parameter estimation and increase the accuracy of the radiation source localization. Meanwhile, this method can obtain the network topology distribution of group targets within the region of interest. The effectiveness of this method has been confirmed through simulation experiments. This method is applicable to distributed systems consisting of three or more receiving stations. If the system contains a very large number of receiving stations, reconfigurable intelligent surface (RIS) technology can be applied to the architecture of the system to improve the data rate [26,27,28].

## 2. Signal Model

In this section, we describe the received signal model for a ground-distributed reconnaissance system for frequency-hopping TDMA airborne payloads.

### 2.1. Transmitted Signal Model

**Figure 1:**
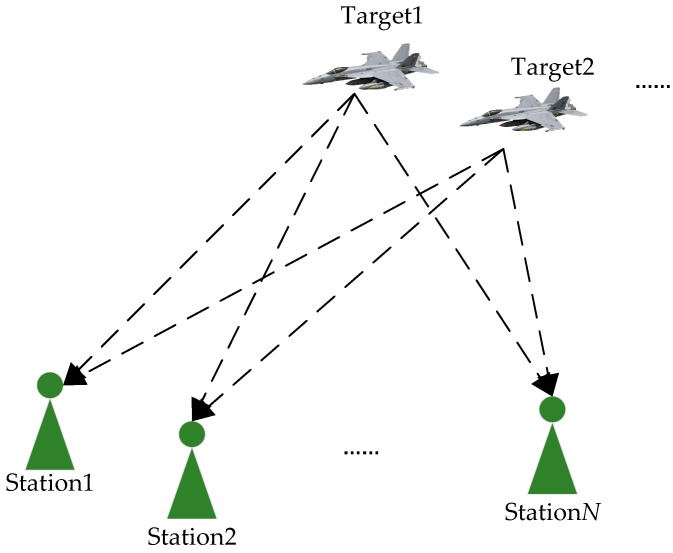
Location scenario of a distributed reconnaissance system.

First, we assume there are multiple airborne moving targets carrying frequency-hopping TDMA communication signal payloads in space. The transmission systems can access different wireless data broadcasting networks according to the task. In addition, the distributed reconnaissance system is composed of *N* stations, and the baseline length between the receiving stations meets the positioning requirements, as shown in Figure 1.

Due to the strong anti-interference ability of communication systems, the pulse carrier frequency of the transmitted signal usually jumps in a pseudo-random sequence. Therefore, taking the transmission signal of a single target as an example, the *k*th hopping pulse can be expressed as

(1) $$x_{k}\left(t\right)=A_{k}r_{k}\left(t\right)e^{j2\pi f_{k}t}+n_{k}\left(t\right),$$

where $A_{k}$ denotes the amplitude of the *k*th hopping pulse, $f_{k}$ is the frequency of the hopping pulse, $n_{k}\left(t\right)$ is Gaussian white noise with a zero mean and a variance of $\sigma_{n}^{2}$, and $r_{k}\left(t\right)$ denotes a rectangular pulse, as follows:

(2) $$r_{k}\left(t\right)=\left\{\begin{array}{l} 1,\;t\in \left[T_{0}+\left(k-1\right)T_{e},T_{0}+\left(k-1\right)T_{e}+T_{p}\right]\\ 0,\;else \end{array}\right.,$$

where $T_{0}$ is the starting time, $T_{e}$ is the period of frequency hopping, and $T_{p}$ is the width of the frequency-hopping pulse. Then, the transmission signal of *M* hops within a single time slot can be represented as

(3) $$x\left(t\right)=\sum_{k=1}^{M}x_{k}\left(t\right).$$

### 2.2. Received Signal Model

The transmission signal passes through the line of sight and reaches the receiving station. After channel calibration at the receiver, the received signal at the *n*th receiving station can be expressed as

(4) $$s_{n}\left(t\right)=a_{n}e^{j\phi_{n}}x\left(t-\tau_{n}\left(t\right)\right)$$

where $a_{n}$ denotes the amplitude attenuation of the signal propagation, $\phi_{n}$ is the initial phase shift of the transmitted signal at the receiving terminal, and $\tau_{n}\left(t\right)$ denotes the propagation delay of the signal from the radiation source to the receiving station. It is assumed that the radiation source target keeps moving at a constant speed during the signal accumulation time. Hence, the delay can be expressed as

(5) $$\tau_{n}\left(t\right)=\frac{d_{n}\left(T_{0}\right)+v_{n}t}{c},$$

where $d_{n}\left(T_{0}\right)$ denotes the distance between the radiation source and the *n*th receiving station at the initial moment, $v_{n}$ denotes the radial velocity of the radiation source relative to the *n*th receiving station, and *c* denotes the speed of light. Then, the TDOA of the radiation source signal received by station $n_{1}$ and station $n_{2}$ can be expressed as

(6) $$\tau_{d}\left(t\right)=\tau_{n_{2}}\left(t\right)-\tau_{n_{1}}\left(t\right)=\tau_{0}+\frac{v_{d}}{c}t,$$

where $\tau_{0}=\left(d_{n_{2}}\left(T_{0}\right)-d_{n_{1}}\left(T_{0}\right)\right)/c$ is the TDOA of the signals received by two receiving stations at the initial time and $v_{d}=v_{n_{2}}-v_{n_{1}}$ is the VDOA of the radiation source relative to two receiving stations. Here, we assume that $n_{1}$ is the reference station. If we substitute Equation (1) into Equation (4), the received signal of station $n_{2}$ can be expressed as

(7) $$\begin{array}{cc} s_{n_{2}}\left(t\right) & =\alpha_{n_{2}}e^{j\phi_{n_{2}}}r_{k}\left(t-\tau_{n_{2}}\left(t\right)\right)e^{j2\pi f_{k}\left(t-\tau_{n_{2}}\left(t\right)\right)}+\eta_{n_{2}}\left(t\right)\\ & =\alpha_{n_{2}}e^{j\phi_{n_{2}}}r_{k}\left(t-\tau_{n_{1}}\left(t\right)-\tau_{d}\left(t\right)\right)e^{j2\pi f_{k}\left(t-\tau_{n_{1}}\left(t\right)-\tau_{d}\left(t\right)\right)}+\eta_{n_{2}}\left(t\right) \end{array},$$

where $\alpha_{n_{2}}=A_{k}\cdot a_{n_{2}}$ is the amplitude of the received signal and $\eta_{n_{2}}\left(t\right)$ is Gaussian stationary white noise with a zero mean.

## 3. Proposed Method

### 3.1. Method Framework

**Figure 2:**
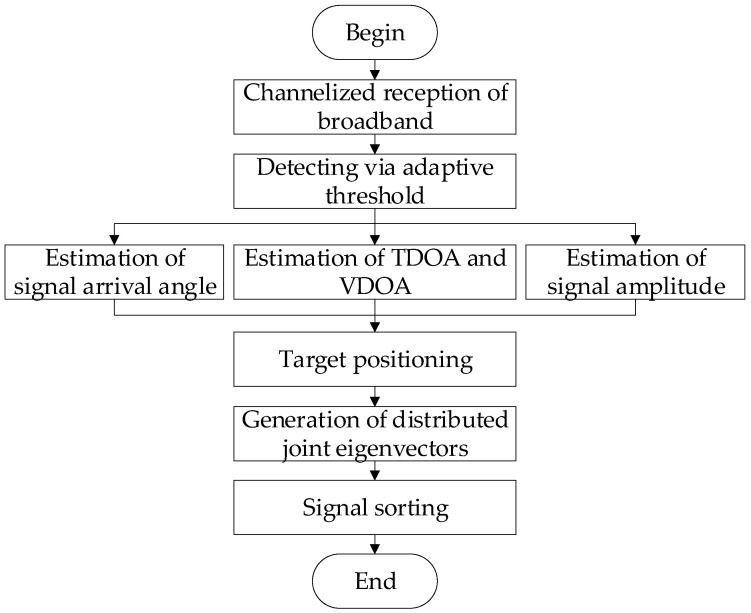
The framework of the proposed method.

Figure 2 shows the framework of the proposed method. Firstly, the signal processing system receives the frequency-hopping signal sent by the antenna front end. Due to the wide frequency band of the signal, wideband channelized reception processing is applied to receive the signal. Then, to separate the frequency-hopping TDMA signals from the noise, the adaptive thresholds are used for point-by-point detection and the parameters of the signals are further measured. After target positioning based on the TDOA and the VDOA, the characteristic parameters of the radiation sources can form the distributed joint eigenvectors. Finally, the feature information of the cluster targets is divided into different networks using the effective clustering algorithm.

### 3.2. Wideband Channelized Reception

**Figure 3:**
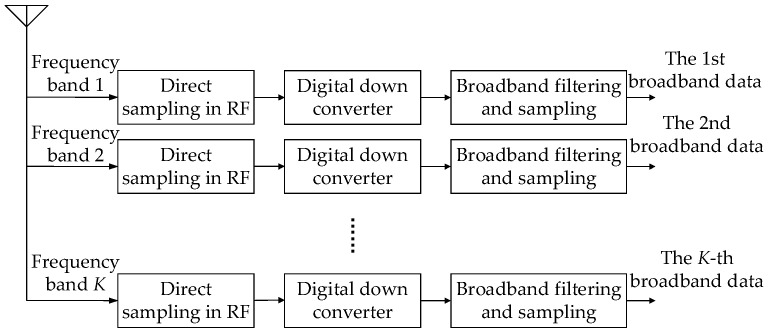
Broadband receiving structure based on RF direct acquisition.

In order to achieve the full probability reception of broadband frequency-hopping TDMA signals, the range of frequency-hopping $\left[f_{L},f_{H}\right]$ is divided into *K*-many sub-bands. The frequency width of each sub-band is $B=\left(f_{H}-f_{L}\right)/K$. As shown in Figure 3, the antenna receives the radiated signal in each frequency band and the signal is sampled directly in radio frequency (RF). The sampled signals are digitally down-converted and filtered according to the sub-bands.

**Figure 4:**
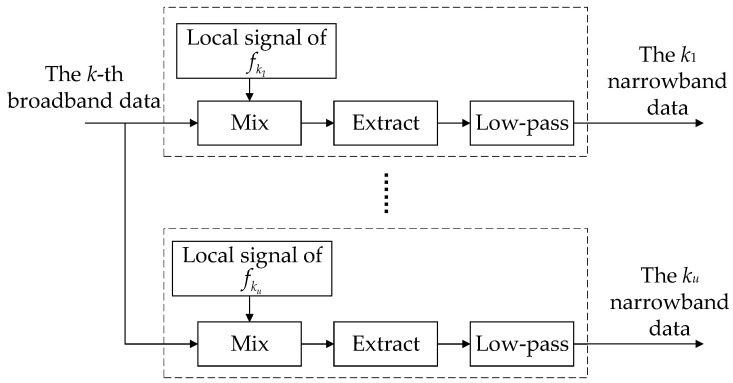
Preprocessing structure based on narrowband mixing.

Then, the narrowband mixing preprocessing is realized via frequency control. The *M* frequency points $\left[f_{1},f_{2},\cdots ,f_{r},\cdots ,f_{M}\right]$ of the TDMA signal are used as the center frequencies of the local oscillator signals, where 1≤r≤M. As shown in Figure 4, the hopping frequency points contained in frequency band *k* are $\left[f_{k_{1}},f_{k_{2}},\cdots ,f_{s},\cdots ,f_{k_{u}}\right]$. The signals extracted via broadband filtering in each frequency band are subjected to frequency mixing preprocessing, where 1≤k≤K, $1\leq s\leq k_{u}$, and $k_{u}$ represents the number of frequencies contained in the *k*th frequency band. In order to further reduce the data rate, the mixed data are extracted and low-pass filtered. According to Equation (7), the narrowband filtering output of the *r*th sub-band of the *n*th station is

(8) $$s_{n,r}\left(t\right)=\beta_{n}e^{j\Theta_{n}}r_{r}\left(t-\tau_{n}\left(t\right)\right)e^{-j2\pi f_{r}\tau_{n}\left(t\right)}+\delta_{n}\left(t\right)$$

where $\beta_{n}$ is the amplitude of the filtered signal and $\delta_{n}\left(t\right)$ is the filtered noise.

### 3.3. Parameter Estimation

From Equation (8), it can be seen that the filtered output contains the target signal and noise. To accurately measure the pulse parameters in a noisy environment, an adaptive threshold detection method is used to obtain a rough measurement of the pulse arrival time. Then, using the matching pulses from each of the two stations, the CAF of coherent accumulation is calculated. By using a two-dimensional spectral peak search, the TDOA and the VDOA can be accurately estimated.

#### 3.3.1. Detection Using an Adaptive Threshold

Firstly, the narrowband filtering output of each sub-band $s_{n,r}\left(t\right)$ is detected using envelope detection. The signal modulus of the *r*th frequency sub-band at the *n*th station, from the starting position at *L* points, is denoted by ${\left\{{\tilde{s}}_{n,r}\left(0,j\right)\right\}}_{j=0}^{j=L-1}$, and its mean is $V_{r}\left(0\right)$. For the signal with a length of *L* in the *h*th segment of the *r*th sub-band, the set $S_{r}\left(h-1\right)$ is composed of values in the signal modulus ${\left\{{\tilde{s}}_{n,r}\left(h-1,j\right)\right\}}_{j=0}^{j=L-1}$ that are greater than the threshold $S_{r}\left(h-2\right)$, where h≥2. The median of set $S_{r}\left(h-1\right)$ is denoted by $\mu_{r}\left(h-1\right)$ and the minimum value is denoted by $\lambda_{r}\left(h-1\right)$. The adaptive iteration factor represents the degree of fluctuation of the signal near the center value, defined as follows:

(9) $$G_{r}\left(h-1\right)=\mu_{r}\left(h-1\right)-2*abs\left(\left(\mu_{r}\left(h-1\right)-\lambda_{r}\left(h-1\right)\right)*ln\left(\varepsilon \right)\right),$$

where ε represents the false alarm probability of threshold detection and ε≪1. Considering adjacent data segments, the signal threshold can be obtained via the weighted averaging of the amplitude mean of the previous signal segment $V_{r}\left(h-2\right)$ with the iteration factor of the subsequent segment obtained from Formula (9), expressed as

(10) $$V_{r}\left(h-1\right)=\alpha *G_{r}\left(h-1\right)+\left(1-\alpha \right)V_{r}\left(h-2\right),$$

where 0<α<1. After obtaining the adaptive threshold, the signal is detected point by point. If there is a signal pulse exceeding the threshold, the arrival time of this pulse is recorded as a roughly measured result of the frequency-hopping moment.

Then, we rearrange the roughly measured hopping moments from small to large. By combining the corresponding frequency points and signal amplitudes, the parameter set can be obtained as

(11) $$T_{n}=\left\{\left(t_{n,1},f_{n,1},\rho_{n,1}\right),\left(t_{n,2},f_{n,2},\rho_{n,2}\right),\cdots ,\left(t_{n,h},f_{n,h},\rho_{n,h}\right),\cdots ,\left(t_{n,N_{n}},f_{n,N_{n}},\rho_{n,N_{n}}\right)\right\},$$

where $t_{n,1}\leq t_{n,2}\leq \cdots t_{n,h}\leq \cdots t_{n,N_{n}}$, $\rho_{n,h}$ is the amplitude of the pulse, and $N_{n}$ is the number of frequency-hopping pulses that pass the threshold. For all elements in the $T_{1}$, $T_{2}$,……, $T_{N}$ signal parameter sets of *N* stations, we compare the pulse frequencies and rough TDOA of each station one by one. If there is

(12) $$\left\{\begin{array}{l} f_{n_{1},m_{1}}=f_{n_{2},m_{2}}\\ abs\left(t_{n_{1},m}-t_{n_{2},m_{2}}\right)<\sqrt{{\left(x_{n_{1}}-x_{n_{2}}\right)}^{2}+{\left(y_{n_{1}}-y_{n_{2}}\right)}^{2}}/c \end{array}\right.,$$

where $\left(x_{n_{1}},y_{n_{1}}\right)$ are the coordinates of the *n*1th station and $\left(x_{n_{2}},y_{n_{2}}\right)$ are the coordinates of the *n*2th station, then the two pulses are considered to be matching pulses, and the start time of the pulses is recorded as $t_{n_{1},m_{1}}$, $t_{n_{2},m_{2}}$.

According to the timeslot characteristics of the TDMA signals, the start time of all matching pulses within the start time is screened. The start time of the first pulse in each time slot is selected to form the sets as follows:

(13) $$T_{n_{1},0}=\left\{{t^{\prime }}_{n_{1},1},{t^{\prime }}_{n_{1},2},\cdots ,{t^{\prime }}_{n_{1},q},\cdots ,{t^{\prime }}_{n_{1},Q}\right\},$$

(14) $$T_{n_{2},0}=\left\{{t^{\prime }}_{n_{2},1},{t^{\prime }}_{n_{2},2},\cdots ,{t^{\prime }}_{n_{2},q},\cdots ,{t^{\prime }}_{n_{2},Q}\right\},$$

where ${t^{\prime }}_{n_{1},q}$ is the starting time of the *q*th time slot of the *n*1th station within the observation time; ${t^{\prime }}_{n_{2},q}$ is the starting time of the *q*th time slot of the *n*2th station within the observation time; and *Q* is the number of time slots, where 1≤q≤Q. By roughly measuring the hopping times of the matching pulses, the search interval of the TDOA can be narrowed. Furthermore, the TDOA and the VDOA can be accurately estimated.

#### 3.3.2. Accurate Estimation of the TDOA and the VDOA

According to Equations (13) and (14), beginning from the pulse start time of the pulse in each time slot between station $n_{1}$ and station $n_{2}$, the narrowband filtered signal of Equation (8) is recorded. The CAF function is used to evaluate the correlation between the received signal and the reference signal, which can also estimate the delay and the Doppler information of the signal. According to the definition of the CAF, the CAF of the *m*th hop signal between station $n_{1}$ and station $n_{2}$ can be expressed as

(15) $$\begin{array}{cc} J_{m}\left(\tau ,v\right) & =\int_{0}^{T_{0}}s_{n1,m}\left(t\right)s_{n2,m}^{*}\left(t+\tau \right)e^{-j2\pi \frac{v}{c}f_{m}t}dt\\ & =\int_{0}^{T_{0}}ue^{j\psi }\rho_{m}\left(t,\tau \right)e^{j2\pi f_{m}\left(-\tau +\tau_{d}\left(t\right)-\frac{v}{c}t\right)}dt\\ & =\int_{0}^{T_{0}}ue^{j\psi }\rho_{m}\left(t,\tau \right)e^{j2\pi f_{m}\left(\left(\tau_{0}-\tau \right)+\left(v_{d}-v\right)\frac{t}{c}\right)}dt \end{array},$$

where $u=\beta_{n_{1}}\cdot \beta_{n_{2}}$, $\psi =\Theta_{n_{1}}-\Theta_{n_{2}}$, and $\rho_{m}\left(t,\tau \right)=r_{m}\left(t-\tau_{n_{1}}\left(t\right)\right)\cdot r_{m}^{*}\left(t+\tau -\tau_{n_{1}}\left(t+\tau \right)-\tau_{d}\left(t+\tau \right)\right)$. It can be seen from the above equation that when $\tau =\tau_{0}$ and $v=v_{d}$, the CAF of the single-hop signal can reach the maximum value.

However, the TDOA and the VDOA estimated using only a single-hop signal cannot meet the positioning requirements. In order to improve the estimation accuracy of the parameter, data within the accumulation time of the W-jumps signal are used to calculate the CAF, where

(16) $$\begin{array}{cc} J\left(\tau ,v\right) & =\int_{0}^{WT_{0}}s_{n1}\left(t\right)s_{n2}^{*}\left(t+\tau \right)e^{-j2\pi \frac{v}{c}ft}dt\\ & =\sum_{m=1}^{W}\int_{0}^{T_{0}}s_{n1,m}\left(t\right)s_{n2,m}^{*}\left(t+\tau \right)e^{-j2\pi \frac{v}{c}f_{m}\left(t+\left(m-1\right)T_{e}\right)}dt\\ & =\sum_{m=1}^{W}J_{m}\left(\tau ,v\right)\cdot e^{-j2\pi \frac{v}{c}f_{m}\left(m-1\right)T_{e}} \end{array}.$$

From the above equation, the phase compensation value of the CAF for the *m*th pulse jump can be obtained as follows:

(17) $$\Theta_{m}=-2\pi \frac{v}{c}f_{m}\left(m-1\right)T_{e}.$$

Therefore, according to Equation (17), the CAF of each hopping $J_{m}\left(\tau ,v\right)$ can be compensated using phase compensation. When $\tau =\tau_{d}$ and $v=v_{d}$, the amplitude of the multi-pulse CAF $\left|J\left(\tau ,v\right)\right|$ reaches its maximum. Thus, a two-dimensional peak search is performed to obtain the estimated results of the TDOA and the VDOA, where

(18) $$\left({\hat{\tau }}_{n1,n2},{\hat{v}}_{n1,n2}\right)=\underset{\tau ,v}{argmax}\left(\left|J\left(\tau ,v\right)\right|\right).$$

### 3.4. Location Estimation Based on the TDOA and the VDOA

In this section, based on the relationship between the distance and the time of signal propagation, we use the TDOA and the VDOA to establish mathematical equations related to the target position. The target position can be obtained by solving these equations.

Under the multi-station positioning model, the distance between the target and the *n*th receiving station can be expressed as

(19) $$r_{n}=\sqrt{{\left(x-x_{n}\right)}^{2}+{\left(y-y_{n}\right)}^{2}}.$$

According to Equation (19), the velocity of the target can be expressed as a derivative of the distance; therefore, we have

(20) $$v_{n}={\dot{r}}_{n}=\frac{dr_{n}}{dt}=\frac{\left(x-x_{n}\right)\dot{x}+\left(y-y_{n}\right)\dot{y}}{\sqrt{{\left(x-x_{n}\right)}^{2}+{\left(y-y_{n}\right)}^{2}}}.$$

According to Equations (19) and (20), the distance difference and the speed difference of the signal arriving at two receiving stations can be expressed as

(21) $$\left\{\begin{array}{l} {\hat{r}}_{n_{1},n_{2}}=\sqrt{{\left(x-x_{n_{2}}\right)}^{2}+{\left(y-y_{n_{2}}\right)}^{2}}-\sqrt{{\left(x-x_{n_{1}}\right)}^{2}+{\left(y-y_{n_{1}}\right)}^{2}}\\ {\hat{v}}_{n1,n2}=\frac{\left(x-x_{n_{2}}\right)\dot{x}+\left(y-y_{n_{2}}\right)\dot{y}}{\sqrt{{\left(x-x_{n_{2}}\right)}^{2}+{\left(y-y_{n_{2}}\right)}^{2}}}-\frac{\left(x-x_{n_{1}}\right)\dot{x}+\left(y-y_{n_{1}}\right)\dot{y}}{\sqrt{{\left(x-x_{n_{1}}\right)}^{2}+{\left(y-y_{n_{1}}\right)}^{2}}} \end{array}\right.,$$

where ${\hat{r}}_{n_{1},n_{2}}={\hat{\tau }}_{n1,n2}\cdot c$, $\left(x,y\right)$ represents the coordinates of the target and $\left(\dot{x},\dot{y}\right)$ is the speed of the target. Two sets of equations can be established using three receiving stations. By substituting ${\hat{\tau }}_{n1,n2}$ and ${\hat{v}}_{n1,n2}$ obtained from Equation (18) into the left-hand side of Equation (21), the position and speed of the target can be solved, which are denoted by $\left(\dot{x},\dot{y}\right)$ and $v_{d}$, where $v_{d}=\sqrt{{\hat{v}}_{x}^{2}+{\hat{v}}_{y}^{2}}$.

### 3.5. Multi-Network Signal Sorting

Due to the possible existence of communication signals from several network radio stations in the received signal, it is necessary to classify the target parameter measurement results obtained above. This is beneficial for understanding the network composition structure and attack patterns of group targets and for tracking specific radio targets.

According to the previously obtained information such as the hopping time, amplitude, position, speed, etc., and combined with the measurement results of the target angle, the distributed joint eigenvector $s_{d}=\left\{t_{d},\rho_{d},x_{d},y_{d},v_{d},\theta_{d}\right\}$ is formed; $d=1,2,\cdots ,N_{c}$, where $N_{c}$ represents the number of pulses. By clustering the samples in the HDW set, each class in the clustering result represents a different network and the sample points contained in each class represent hopping signals belonging to the same class.

Assuming that the sample set $\left\{s_{1},s_{2},\cdots ,s_{N_{c}}\right\}$ is divided into *U* classes, the *u*th class sample can be expressed as $\left\{s_{d}^{u};d=1,2,\cdots ,n_{u};u=1,2,\cdots ,U\right\}$. $n_{u}$ represents the number of samples contained in the *u*th class and ${\sum_{u=1}^{U}n_{u}=N}_{c}$. The centroid of the *u*th class can be expressed as

(22) $$\mu_{u}=\frac{1}{n_{u}}\sum_{d=1}^{n_{u}}s_{d}^{u}.$$

The clustering criterion function defined according to the minimum intra-class distance can be expressed as follows [29]:

(23) $$E=min\left\{\sum_{u=1}^{U}\sum_{d=1}^{n_{u}}{\left\|s_{d}^{u}-\mu_{u}\right\|}^{2}\right\}.$$

The number of classes after the initial clustering is set as $K_{p}$ ($K_{p}>\sqrt{N}$). Sample set $\left\{s_{1},s_{2},\cdots ,s_{N_{c}}\right\}$ is clustered via the K-means method and is classified into $K_{p}$ categories. The steps are as follows:

(1) Randomly select $K_{p}$ samples as the initial cluster centers;
(2) Obtain the distance between each sample and the cluster center, and divide it into the class with the closest distance;
(3) Calculate and update the corresponding cluster center value of each category;
(4) Repeat the above process until the cluster center no longer changes.

Then, we use the AHC (agglomerative hierarchical clustering) algorithm to merge the $K_{p}$ samples. The steps are as follows:

(1) Treat each data point in the data set as a class;
(2) According to the cosine similarity method, solve the similarity of all classes;
(3) Obtain the class with the closest similarity and classify the two into one class;
(4) Repeat the above process until the number of clusters is *U*.

*U* network feature sets can be obtained via clustering. Each feature set represents a network group. The feature vector in each feature set contains the feature information belonging to a single target in the same network.

## 4. Numerical Results

This section describes the verification of the performance of the proposed method via simulation. A distributed passive reconnaissance system with three receiving stations was simulated. The coordinates of the stations were $\left(0\;\mathrm{km},\;0\;\mathrm{km}\right)$, $\left(1.17\;\mathrm{km},\;10.22\;\mathrm{km}\right)$, and $\left(1.17\;\mathrm{km},\;20.83\;\mathrm{km}\right)$. In addition, the sampling frequency of the system was 240 MHz. The signal of the radiation source was in the form of frequency-hopping TDMA. The signal parameters are shown in Table 1. The SNR was 5 dB and the noise was Gaussian white noise.

There were 18 targets in the simulation space belonging to five different network groups. The radiation source signals within each network group were synchronous pseudo-random frequency hopping, and the signals from different network groups intersected and wove. The coordinates and velocities of the radiation sources in different scenarios are shown in Table 2.

**Figure 5:**
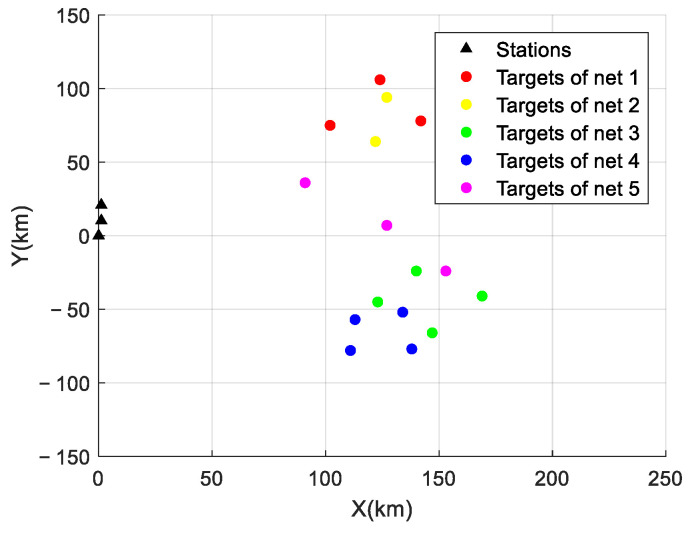
Experimental scenario.

The distribution of the initial positions of the reconnaissance stations and the radiation sources is shown in Figure 5.

### 4.1. Experiment 1

**Figure 6:**
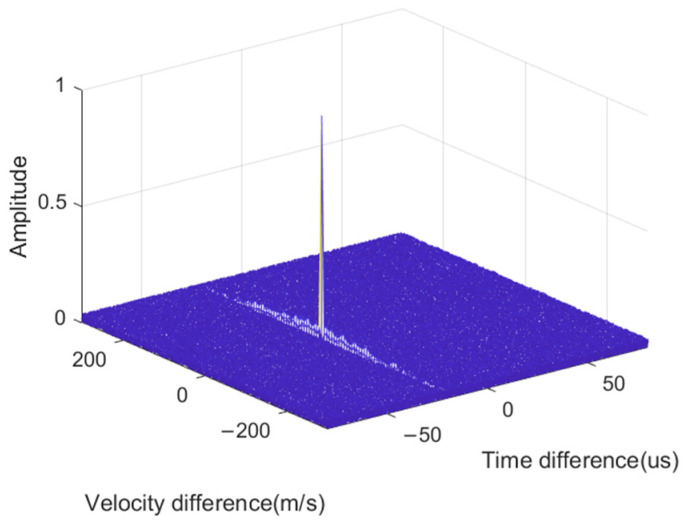
Coherent accumulation peak of CAF.

The received signals of the three stations were processed using the proposed algorithm and 300 pulses within a single time slot were accumulated. The normalized result of the mutual ambiguity function accumulation between the main station and the first substation is shown in Figure 6. The proposed method formed sharp peaks of the CAF and the accumulated SNR was high. This demonstrates the obvious noise resistance advantage of the proposed method.

**Figure 7:**
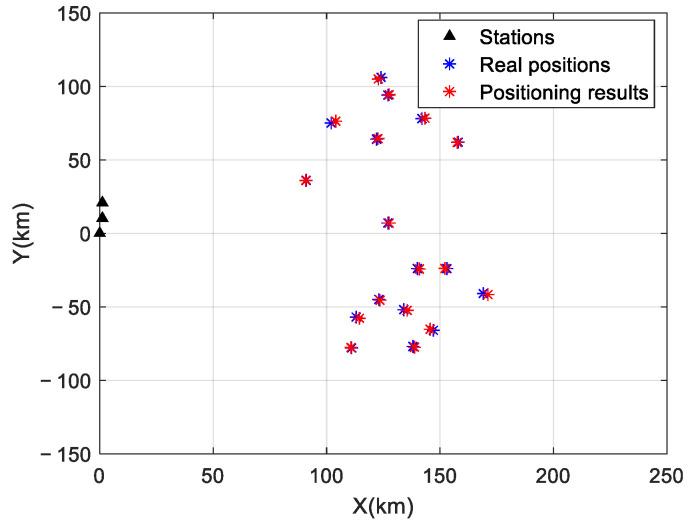
Target positioning results.

The target positioning results based on the TDOA/VDOA are shown in Figure 7. Under an SNR of −5 dB, the proposed algorithm formed positioning points near the true positions of the targets. The positioning effect of the algorithm was not affected by the signal mixing of multiple network stations, which proved the positioning effectiveness of the proposed method under low SNR conditions.

As shown in Table 3, we calculated the error of the TDOA, the error of the VDOA, and the error of the positioning of each target within a single time slot. The symbols $\Delta \tau_{1,2}$ and $\Delta \tau_{1,3}$ represent the error of the TDOA between the main station and the first substation, and the error of the TDOA between the main station and the second substation, respectively. The symbols $\Delta v_{1,2}$ and $\Delta v_{1,3}$ represent the error of the VDOA between the main station and the first substation, and the error of the VDOA between the main station and the second substation. The measurement error of the algorithm for the TDOA and the VDOA was close to the theoretical value, and the positioning error was less than 0.4%R.

**Figure 8:**
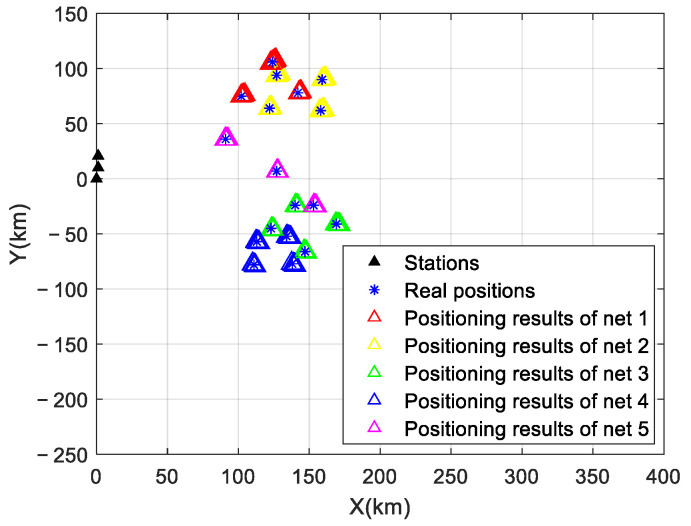
Signal sorting results.

The signal sorting results are shown in Figure 8. In the case of signal aliasing, the signals from five networks were correctly sorted without any outliers.

### 4.2. Experiment 2

**Figure 9:**
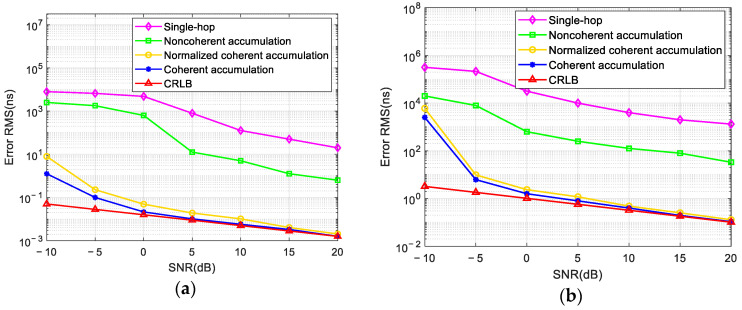
Comparison of the parameter estimation accuracy among various algorithms: (**a**) the TDOA error; (**b**) the VDOA error.

As shown in Figure 9a,b, we compared the relationship between the error of the TDOA, the error of the VDOA, and the SNR obtained using the single-hop method, the noncoherent accumulation method, the normalized coherent accumulation method in [9,10,11], and the coherent accumulation method proposed in this paper and then compared them with the CRLB (Cramer–Rao Lower Bound) value. The SNR increased with a step size of 5 dB between −10 dB and 20 dB. The number of Monte Carlo experiments was 200. As shown in the figures, with the increase in the SNR, the parameter estimation error of the method proposed in this paper converged quickly, and the parameter estimation accuracy was always higher than the other methods. When the SNR was higher than 0 dB, the proposed method and the normalized coherent accumulation method approached the value of the CRLB, while the single-hop method and noncoherent accumulation method could not achieve the ideal errors for the parameter estimation.

### 4.3. Experiment 3

**Figure 10:**
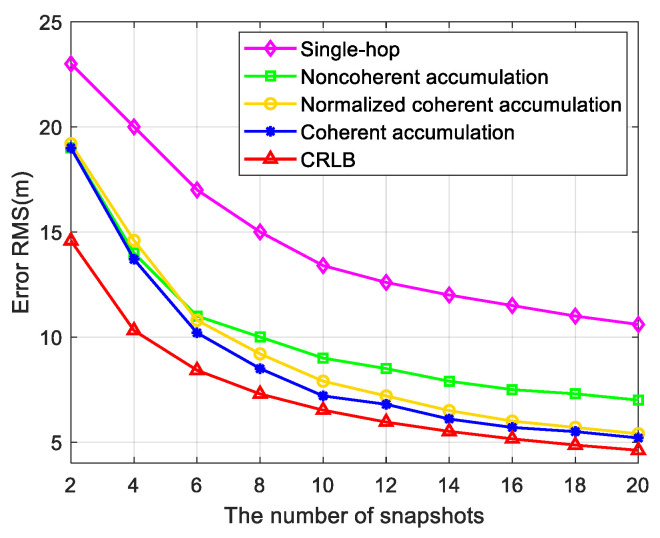
Comparison of the positioning accuracy of different algorithms.

As shown in Figure 10, we compared the relationship between the positioning error and the number of snapshots among the single-hop method in [8], the noncoherent accumulation method, the normalized coherent accumulation method in [9,10,11], and the coherent accumulation method proposed in this paper and then compared them with the CRLB value. The number of snapshots increased with a step size of two within the range [2,20], and the number of Monte Carlo experiments was 200. As shown in the figure, when the number of snapshots was less than or equal to four, the positioning error of our method was similar to that of the noncoherent accumulation method and the normalized coherent accumulation method. However, as the number of snapshots increased, the positioning error of the proposed method gradually converged to the vicinity of the CRLB. Compared with the other methods, the proposed method had a faster convergence speed and a higher positioning accuracy.

### 4.4. Experiment 4

**Figure 11:**
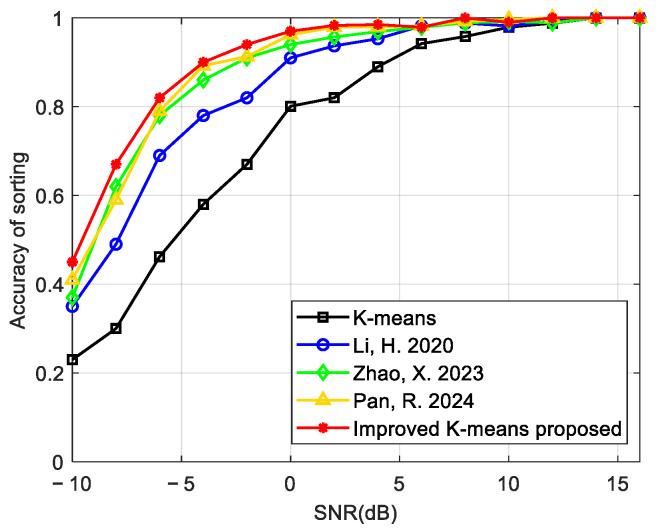
Comparison of the sorting accuracy of the K-means algorithm, the improved K-means algorithms in [17,18,19], and the improved K-means method proposed in this paper.

As shown in Figure 11, we analyzed the relationship between the sorting accuracy and the SNR among the K-means algorithm, the improved K-means algorithms in [17,18,19], and the improved K-means method proposed in this paper. The SNR increased with a step size of 2 dB between −10 dB and 16 dB, and the number of Monte Carlo experiments was 200. Compared with the current K-means algorithm, the improved K-means algorithm in this paper had a higher sorting accuracy and a faster convergence speed. When the SNR was greater than or equal to −5 dB, the sorting accuracy of the algorithm in this paper was greater than 0.9, thus achieving a high accuracy of sorting in a low SNR environment.

## 5. Conclusions

Taking the mixed multi-station frequency-hopping TDMA signal as the research object, this paper presented a distributed passive localization and network-sorting method based on two-level parameter estimation and joint clustering. This method combines adaptive threshold detection with the coherent accumulation of the CAF, achieving the accurate measurement of signal TDOA and VDOA under a low sample condition. Moreover, a clustering and sorting method was designed based on the distributed joint eigenvectors of multi-stations, which can accurately distinguish signals from multi-network stations in the case of aliasing. The simulation experiment results show that the method proposed in this paper is more accurate in locating mixed signals. In addition, it has better positioning and sorting capabilities under a low SNR and low snapshot conditions.

## Figures and Tables

**Table 1:** Signal parameters.

Parameters	Numerical Values
Length of a single time slot	3.9 ms
Number of data pulses	300
Width of a pulse	6.4 µs
Interval between adjacent pulses	13 µs
Symbol rate	5 MHz
Interval between adjacent pulses	$\left[900\;\mathrm{MHz},\;1000\;\mathrm{MHz}\right]$
Frequency-hopping interval	5 MHz
Frequency points	21

**Table 2:** Coordinates and velocities of the radiation sources.

Network Number	Coordinates of Radiation Sources (km)	Velocities of Radiation Sources (m/s)
1	(102, 75), (124, 106), (142, 78)	270, 300, 260
2	(127, 94), (159, 90), (122, 64), (158, 62)	280, 220, 310, 240
3	(123, −45), (140, −24), (147, −66), (169, −41)	−290, −285, −280, −300
4	(113, −57), (111, −78), (134, −52), (138, −77)	260, 240, 270, 280
5	(91,36), (127,7), (153, -24)	−245, −250, −230, −220

**Table 3:** Statistics of the positioning errors.

Radiation Source	1	2	3	4	5	6	7	8	9
$\Delta \tau_{1,2}$ (ns)	0.20	0.14	0.07	0.01	0.12	0.18	0.13	0.04	0.13
$\Delta \tau_{1,3}$ (ns)	0.04	0.09	0.20	0.15	0.15	0.10	0.03	0.20	0.10
$\Delta v_{1,2}$ (m/s)	6.95	10.45	6.83	8.48	5.82	7.88	3.43	4.10	7.52
$\Delta v_{1,3}$ (m/s)	3.50	5.87	4.52	8.79	6.54	8.43	9.84	8.61	10.70
position error (%R)	0.16	0.07	0.08	0.16	0.04	0.37	0.05	0.18	0.03
Radiation source	10	11	12	13	14	15	16	17	18
$\Delta \tau_{1,2}$ (ns)	0.12	0.16	0.17	0.07	0.08	0.20	0.09	0.11	0.18
$\Delta \tau_{1,3}$ (ns)	0.03	0.20	0.09	0.13	0.10	0.08	0.11	0.10	0.05
$\Delta v_{1,2}$ (m/s)	3.52	8.27	7.94	5.82	11.20	6.37	4.56	9.57	7.91
$\Delta v_{1,3}$ (m/s)	8.60	4.66	4.37	6.77	5.38	7.82	2.82	3.58	5.89
position error (%R)	0.05	0.04	0.22	0.14	0.35	0.08	0.23	0.11	0.09

## Data Availability

Data are contained within the article.
